# Seronegative Granulomatosis With Polyangiitis With Isolated Lung Lesions: A Case Report

**DOI:** 10.7759/cureus.59692

**Published:** 2024-05-05

**Authors:** Harkesh Arora, Dhiran Sivasubramanian, Sinduja Sivakumar, Aditya Sambandam, Sreekant Avula

**Affiliations:** 1 Hospital Medicine, Lovelace Medical Center, Albuquerque, USA; 2 Internal Medicine, Coimbatore Medical College, Coimbatore, IND; 3 Medicine, Burrell College of Osteopathic Medicine, Las Cruces, USA; 4 Medicine, University of New Mexico School of Medicine, Albuquerque, USA; 5 Internal Medicine, University of Minnesota, Minneapolis, USA

**Keywords:** wegener’s granulomatosis, pulmonary disease, pulmonary, clinical rheumatology, lung lesions, polangiitis, seronegative granulomatosis

## Abstract

Granulomatosis with polyangiitis (GPA), previously referred to as Wegener's granulomatosis, is an uncommon form of necrotizing vasculitis that predominantly targets small and medium-sized blood vessels as a result of granulomatous inflammation. Granulomatosis with polyangiitis is defined by the existence of necrotizing granulomas in the upper respiratory tract, along with renal involvement, which includes necrotizing glomerulonephritis with extra capillary crescents. From a diagnostic perspective, there is a high correlation between GPA and proteinase-3 anti-neutrophil cytoplasmic antibody (PR3-ANCA) because of the release of inflammatory cytokines, reactive oxygen species (ROS), and lytic enzymes. While ANCA-positive serology is commonly used as the diagnostic criteria, we present a seronegative GPA case with isolated lung lesions. A 54-year-old woman was referred for an assessment of hemoptysis and alterations in her chest radiograph. The patient's laboratory results showed a positive QuantiFERON test but negative results for ANCA and antinuclear antibodies (ANA) tests. A chest CT scan showed the presence of several pulmonary nodules in both lungs, with some cavitation. A CT-guided biopsy was conducted on a nodule located in the lower lobe of the right lung. The results showed that the nodule had non-neoplastic chronic inflammation and an area of geographic necrosis. A second robotic-assisted left upper and lower lobe wedge resection was done, which showed white to tan granular lesions with necrotizing granulomatous inflammation and lymph nodes with anthracosis and a lot of histiocytes, which is typical of GPA. The patient received a six-month course of intravenous rituximab treatment.

## Introduction

Granulomatosis with polyangiitis (GPA), or Wegener’s granulomatosis, is a rare necrotizing vasculitis caused by granulomatous inflammation affecting primarily small and medium blood vessels. Granulomatosis with polyangiitis is characterized by necrotizing granulomas of the upper respiratory tract with renal involvement, including necrotizing glomerulonephritis with extra capillary crescents [1]. Patients can additionally present with otologic manifestations, occurring in roughly 20%-70% of cases [2]. The etiology of GPA is unknown, with some cases attributed to dust inhalation, silica exposure, and other potentially infectious agents [3]. Diagnostically, GPA is strongly associated with proteinase-3 anti-neutrophil cytoplasmic antibody (PR3-ANCA) due to the release of inflammatory cytokines, reactive oxygen species (ROS), and lytic enzymes [4]. Though ANCA-positive serology is the widely accepted diagnostic criteria, we present a case of seronegative GPA with isolated lung lesions.

## Case presentation

A 54-year-old female was referred for evaluation of hemoptysis, and an abnormal cavity was noticed on the chest radiograph. The patient presented with a worsening cough with blood-streaked sputum for the past few months. She denied any history of dyspnea, chest pain, wheezing, or fever. She denied any history of asthma, chronic obstructive pulmonary disease, lung mass, or pneumonia. The patient had no history of weight loss or appetite changes. The patient was admitted to frequent sinus infections and bronchitis and has been on antibiotics six times in the last two years, and her symptoms improved without complete resolution. The patient reported a history of positive purified protein derivative (PPD) test. She denied any emergency room, urgent care visits, or hospitalization for these infections. There was no history of intubation or mechanical ventilation. She reported working as an electrician for eight years but denied any recent travel history or animal exposure concerning pulmonary diseases. She was a former cigarette smoker with five pack years and quit four years before the presentation. She did not report any significant family history. Her current medications included levothyroxine 100 mcg, meloxicam 7.5 mg, and ranitidine 300 mg.

On physical examination, her temperature was 37.3°C, her heart rate was 85 beats per minute, her respiratory rate was 16 breaths per minute, her blood pressure was 126/77 mmHg, and her oxygen saturation was 94% on room air. Organ system-oriented physical examination was normal.

**Figure 1:**
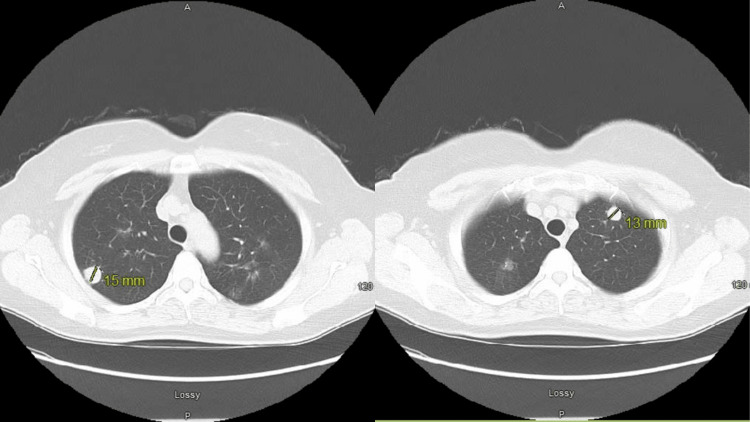
Computed tomography of the thorax revealed multiple bilateral pulmonary nodules with some areas of cavitation. The larger solid nodule measures 15 mm in size in the right upper lobe and 13 mm in the left lung apex.

Routine blood investigations were within the reference range, except for a positive QuantiFERON test. There was no leukocytosis or anemia. Renal and liver function tests were normal. The chest radiograph done in the previous hospital showed an 8-mm cavitary right upper lobe lung lesion. Computed tomography of the thorax revealed multiple bilateral pulmonary nodules with some areas of cavitation, with the largest solid nodule measuring 13 mm in the left lung apex and 15 mm in the right upper lobe. There were no pathologically enlarged mediastinal, hilar, or axillary lymph nodes (Figure 1).

**Figure 2:**
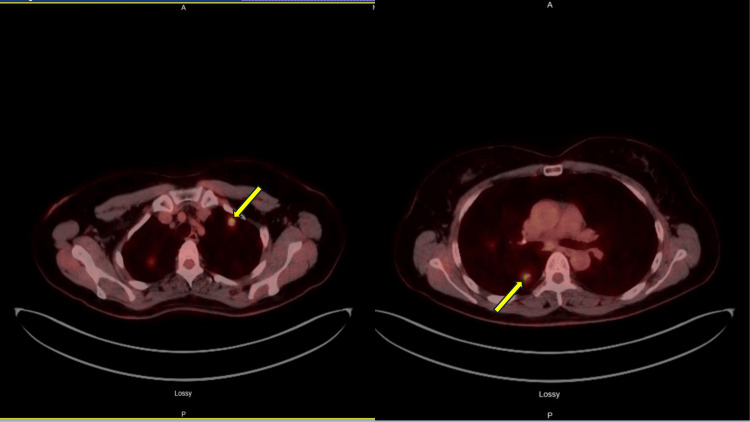
A PET-CT revealed multiple moderately hypermetabolic pulmonary nodules in the upper lobes, predominantly of both lungs (solid yellow arrow). PET-CT: positron emission tomography-computed tomography

With these clinical and radiological findings, the differential diagnosis included granulomatous lung disease (sarcoidosis), ANCA-related inflammatory nodules, atypical mycobacterium infection secondary to a positive QuantiFERON test, and coccidioidomycosis. The sputum examination for acid-fast bacilli (AFB) stain and culture for three samples was unremarkable. A CT-guided biopsy of a right lung lobe pulmonary nodule was performed, which was inconclusive. Bronchioalveolar lavage was performed through bronchoscopy. The AFB stain and culture came negative for both CT-guided biopsy and bronchoscopy samples. A PET-CT revealed multiple moderately hypermetabolic pulmonary nodules with upper lobe predominance in both lungs (Figure 2).

Some of the lesions were cavitating, but there was no evidence of any primary hypermetabolic malignancy. The ANCA and anti-nuclear antibody (ANA) tests were also negative. A repeat CT-guided biopsy was performed on a right lower lobe pulmonary nodule. The pathology results showed non-neoplastic chronic inflammation with an area of geographic necrosis and were negative for AFB and periodic-acid Schiff (PAS) stains. No granulomas, viral inclusion, or evidence of malignancy was visualized. The serology for collagen diseases was negative. A robotic-assisted left upper lobe and left lower lobe wedge resection with mediastinal lymph node dissection was performed. The specimens were sent for histopathological examination. The pathology revealed two indurated, white-to-tan granular lesions with central cavitation. On histological examination, lung parenchyma showed necrotizing granulomatous inflammation and lymph nodes revealed anthracosis and abundant histiocytes. No fungal, mycobacterial, or bacterial organisms were identified in PAS, AFB, or Gram stains.

The possible differential diagnoses considered were pulmonary tuberculosis since the QuantiFERON gold test was positive, fungal pneumonia, atypical *Mycobacterium pneumonia*, sarcoidosis, and GPA. The absence of caseating granuloma, negative AFB stain, and culture ruled out pulmonary tuberculosis. Sarcoidosis was ruled out since there were no non-necrotizing, non-caseating granulomas, and similarly, the fungal granuloma was ruled out as fungal cultures were negative.

According to the criteria established by the American College of Rheumatology for the diagnosis of GPA, the presence of two or more of these four criteria was associated with an 88% sensitivity and 92% specificity: (i) urinary sediment containing red blood cell casts or more than five red blood cells per high-power field; (ii) abnormal findings on the chest radiograph; (iii) oral ulcers or nasal discharge; and (iv) granulomatous inflammation on biopsy. Our patient satisfied two of the above criteria; a diagnosis of seronegative limited granulomatosis with polyangiitis without renal involvement was made. After consultation with a rheumatologist, the patient was started on treatment with rituximab, an anti-CD20 B-cell-depleting monoclonal antibody. The treatment is given in two phases: the induction phase and the maintenance phase. Intravenous rituximab (375 mg) was administered once every week for one month and then once every month for six months. Subsequent follow-ups demonstrated improvement in symptoms. A follow-up chest CT scan after four months of treatment showed no new lesions. Previously noted solid and cavitary nodules substantially reduced size or complete resolution.

## Discussion

Friedrich Wegener first identified GPA as a distinct condition in 1936, dubbed "Wegener's granulomatosis." The syndrome can manifest in "limited forms" involving the middle and inner ears, the subglottic area of the larynx, the nose, and the paranasal sinuses [5]. Granulomatosis with polyangiitis is a rare necrotizing vasculitis caused by granulomatous inflammation, primarily affecting small and medium blood vessels. It is characterized by the presence of necrotizing granulomas of the upper respiratory tract with renal involvement, including necrotizing glomerulonephritis with extra capillary crescents, and a strong association with PR3-ANCA. The exact cause of GPA is still mostly unknown, but it is thought to be of autoimmune origin and is triggered by environmental factors in those with a genetic predisposition. Environmental factors, including dust inhalation or silica exposure, are probably implicated but only appear in 10% of patients with GPA. Granulomatosis with polyangiitis is associated with a high mortality rate if left untreated. Early diagnosis and treatment are critical. It can manifest in individuals from all racial groupings; however, it primarily impacts individuals of Caucasian descent. Both genders are equally impacted. 

Granulomatosis with polyangiitis is defined by the presence of necrotizing granulomatous lesions in the respiratory system, as well as vasculitis and glomerulonephritis. The upper respiratory tract is affected in 70%-100% of instances with GPA, and traditional symptoms related to the ear, nose, and throat (ENT) may be the initial clinical signs of the disease. The nasal cavity and paranasal sinuses are frequently affected in the head and neck region, with a prevalence of 85%-100% [6]. In contrast, otological disease is present in around 35% of cases, with a range of 19%-61% [5]. Signs of ENT conditions include the presence of crusty nasal discharge, sinus inflammation, a long-lasting middle ear infection, or facial cartilage damage leading to deformities such as a saddle-nose appearance with a scooped-out or sunken nose. Additionally, there may be perforation of the nasal septum, palate, or the outer part of the ear [3]. Nearly 90% of patients have pulmonary involvement, and 80% are said to have renal involvement [7]. There have been reports of cases where the kidneys were spared from minimal GPA [4]. In 16 recorded cases of GPA, the illness was localized to the lungs, and no other organs, including the kidneys, were affected [8]. Additionally, a single case report of restricted GPA did not include the upper respiratory tract or the kidneys [9].

Our patient presented with hemoptysis and cavitatory lesions in the lung on the chest radiograph. The patient's extensive workup done for the evaluation of the cavitatory lesion was negative for tuberculosis and other granulomatous diseases, as well as negative serology. Clinical evaluation, serological testing for ANCA, and histological examination all contribute to diagnosing GPA. The expected 10-year survival rate is 40% when the kidneys are affected and 60%-70% when there is no renal involvement. According to the criteria established by the American College of Rheumatology for the diagnosis of GPA, the presence of two or more of these four criteria was associated with an 88% sensitivity and 92% specificity: (i) urinary sediment containing red blood cell casts or more than five red blood cells per high-power field; (ii) abnormal findings on the chest radiograph; (iii) oral ulcers or nasal discharge; and (iv) granulomatous inflammation on biopsy [10]. As our patient satisfied two of the above criteria, a diagnosis of seronegative limited granulomatosis with polyangiitis without renal involvement was made. It is important to note that confirmation of GPA by ANCA serology is unnecessary to rule out other forms of systemic vasculitis or vasculitis mimics; a diagnosis of GPA should be based on clinical and histological evidence [11, 12].

A timely diagnosis of GPA is crucial for prognostic purposes, as immunosuppressive treatments can rapidly induce clinical remission and ultimately decrease the morbidity and mortality associated with the disease. It is important to note that a positive ANCA serology is unnecessary for diagnosing GPA if sufficient clinical and histological evidence supports the diagnosis. It is essential to use caution when interpreting positive ANCA serology results without clinical indications, symptoms, or histological data that imply ANCA-associated vasculitides.

The extent of the disease determines the management of GPA. The localized form of GPA has a highly favorable outlook. Granulomatosis with polyangiitis is treated in two distinct phases: induction and maintenance. Traditionally, cyclophosphamide, in conjunction with pulse dosage steroids, is employed for induction in severe manifestations of the ailment. Rituximab, a monoclonal antibody that depletes B-cells by targeting CD20, is just as effective as cyclophosphamide for initial treatment and may even be more effective for preventing recurrence. It is currently the preferred therapeutic option [13].

It is usual practice to employ methotrexate and steroids for induction in patients with moderate to mild illnesses. Pulmonary hemorrhage, worsening respiratory compromise, fulminant illness, and rapidly deteriorating kidney function are all indications of plasmapheresis [14]. Maintenance therapy starts three to six months after the patient has recovered. The maintenance medications utilized include methotrexate, rituximab, and azathioprine. The safety profile of azathioprine is the best, and steroids are gradually tapered off [11, 14]. After consultation with a rheumatologist, our patient was started on IV rituximab (375 mg) once every week for one month and then once every month for six months. Symptoms were found to have improved at subsequent follow-up appointments. Four months after starting treatment, a chest CT scan revealed no new lesions; the solid and cavitary nodules that had been previously identified had either shrunk in size or gone entirely as a result of the medication.

## Conclusions

Granulomatosis with polyangiitis is associated with an all-cause mortality rate of 80% in untreated patients. Early diagnosis and treatment are critical. While ANCA serology is a widely accepted diagnostic criterion, this case highlights the importance of having a high index of suspicion without the presence of ANCA as an indicative serological marker. In this case, our patient presented with isolated lung lesions, leading to a better prognosis and response to treatment. Although ANCA is a highly predictive marker for GPA, in patients presenting with cavitary lung lesions with negative ANCA, GPA should still be suspected to improve patient outcomes.
